# Review of suicidal ideation during pregnancy: risk factors, prevalence, assessment instruments and consequences

**DOI:** 10.1186/s41155-022-00220-4

**Published:** 2022-05-24

**Authors:** Pilar Carolina Castelao Legazpi, María F. Rodríguez-Muñoz, María Eugenia Olivares-Crespo, Nuria Izquierdo-Méndez

**Affiliations:** 1 Department of Psychology, Universidad Nacional de Educación a Distancia (UNED), Madrid, Spain; 2 Deparment of Gynecology and Obstetrics, Hospital Clínico San Carlos & Faculty of Medicine Universidad Complutense de Madrid, Madrid, Spain

**Keywords:** Suicidal ideation, Pregnancy, Prevention, Risk factors, Prevalence

## Abstract

**Background:**

Pregnancy is a period when women are particularly vulnerable to suicidal ideation and a great opportunity for suicide risk prevention.

**Aims:**

This study aimed to establish a comprehensive understanding of suicidal ideation prevalence, risk factors, screening tools, consequences and management during pregnancy.

**Method:**

A literature search was performed in MEDLINE and PsycInfo databases from 2016 to 2021. A narrative synthesis of the literature and a critical overview of the current issues/questions to be addressed within the topic of suicidal ideation during pregnancy was performed.

**Results:**

The prevalence of suicidal ideation during pregnancy was between 2.73 and 18% internationally. The risk factors identified were major depressive disorder, anxiety disorder, difficulties with sleep, previous suicide attempts, high rumination, low incomes, being black, being young, low educational level, partner violence, having poor support, food insecurity, history of child abuse, high obstetric risk, multiparity, previous induced abortion and exposure to tobacco or human immunodeficiency virus diagnosis. The screening tools used for suicidal ideation during pregnancy were item 10 of the Edinburgh Postpartum Depression Scale and item 9 of the Patient Health Questionnaire. Results showed that suicidal ideation during pregnancy is associated with poor cognitive development in children and low birth weight. No case management studies on suicidal ideation were found.

**Limitations:**

The main limitation of the available studies was the lack of articles with a high degree of methodological rigour on this subject.

**Conclusions:**

This narrative review is a state-of-the-art paper about suicidal ideation during pregnancy. Further research is needed, and researchers should carry out systematic reviews and meta-analyses, leading to Clinical Practice Guidelines in this area. This effort would improve our evidence-based practice in Perinatal Psychology and prevent associated suicidal behaviour.

## Introduction

Research on suicide has been blocked for many decades (Goodfellow et al., 2020). According to the World Health Organization (2014), ‘suicidal behaviour is understood as a diversity of behaviour that includes thinking about suicide (or suicidal ideation), planning suicide, attempting suicide and committing an act of suicide’. Suicidal ideation is defined as thoughts of committing suicide-related behaviour (O'Carroll et al., 1996). Preventive suicide interventions are needed, especially considering the consequences for families and children who lose their mothers for this reason (Lysell et al., 2018).

Therefore, we must understand the available evidence on suicidal ideation during pregnancy. Pregnancy is when women are particularly vulnerable to mental health problems, including suicidal ideation (Szpunar et al., 2020). During pregnancy, the prevalence of suicidal ideation varies amongst studies from 2.6 to 29.2% (Copersino et al., 2008; Gavin et al., 2011; Lindahl et al., 2005; Melville et al., 2010; Newport et al., 2007). Depression has been established as a risk factor for suicidal ideation during pregnancy (Gavin et al., 2011; Rodriguez et al., 2018). The epidemiologic review by Gelaye et al. (2016) also showed that the risk factors for suicidal ideation during pregnancy include intimate partner violence, less than 12 years of education and major depressive disorder. Suicidal ideation is generally assessed using the Edinburgh Postnatal Depression Scale (EPDS) or the Patient Health Questionnaire (PHQ-9; Al-Halabi et al., 2019). Antepartum suicidal ideation is associated with consequences such as a myriad of adverse maternal and infant outcomes (Copersino et al., 2008; Gavin et al., 2011; Lindahl et al., 2005). The case management of suicidal ideation during pregnancy has not been studied well. However, the Department of Psychiatry at Columbia University and The New York Psychiatric Institute developed the ‘Assess, Intervene, Monitor for Suicide Prevention’ which is a procedure to assess, intervene and monitor suicide prevention in the general population (Brodsky et al., 2018; Labouliere et al., 2018; Stanley & Brown, 2012). The Australian Centre of Perinatal Excellence (COPE) also has a guideline on mental healthcare during the perinatal period which provides various actions for suicide risk prevention (Centre of Perinatal Excellence [COPE]., 2017). Nevertheless, we need additional information on suicidal ideation during pregnancy regarding prevalence, risk factors, consequences, instruments for detection and management strategies. The research focused on suicidal ideation during pregnancy is still lacking. A global understanding of this topic may help prevent and manage efforts of this clinical condition.

To our knowledge, only a few epidemiologic reviews (Gelaye et al., 2016), systematic reviews (O'Connor et al., 2018; Xiao et al., 2022) or meta-analyses (Orri et al., 2019) on suicidal ideation during pregnancy have been conducted on specific topic. Research on suicidal ideation during pregnancy is preliminary because perinatal suicide is a taboo subject. The available evidence is also disjointed. We need to synthesize knowledge to move forward from an overview of what is going on which factor underlines the need for a narrative review to be carried out. Therefore, this narrative review aimed to update the current knowledge on suicidal ideation during pregnancy regarding prevalence, risk factors, assessment instruments, consequences and case management.

## Methods

This paper reported findings from a comprehensive narrative synthesis of previously published international results about suicidal ideation during pregnancy. The purpose was to identify studies that reveal current knowledge on this topic.

In line with Siddaway’s ‘Practice Guide for Conducting and Reporting Narrative Reviews, Meta-Analyses and Meta-Syntheses’ (2018), systematic reviews were characterized by a methodical and replicable methodology and presentation. These reviews may be a systematic review of quantitative (meta-analysis) or qualitative (narrative review, meta-synthesis) information. Given that published work on suicidal ideation during pregnancy is scarce, a narrative review of the issue was considered pertinent. We used the term ‘narrative review’ to refer to an attempt to summarize the literature in an inexplicitly systematic way (Higgins & Green, 2022). A narrative review would be appropriate when a literature review is desired about a collection of quantitative studies that used diverse methodologies or examined different theoretical conceptualizations, constructs and/or relationships (Baumeister, 2013), as in this case. Following Baethge et al.’s (2019) approach, making good quality narrative reviews is desirable. Whilst systematic reviews are superior to narrative reviews in answering specific questions (e.g. whether changing the psychotherapeutic approach with women with suicidal ideation is advisable if she is not responding to the current one), narrative reviews are better suited to addressing a topic in wider ways (e.g. outlining the general principles of identification and manage cases in suicidal ideation during pregnancy).

Sánchez-Meca and Botella (2010) underlined that using Internet resources and critical analysis is required to perform an operational search. Hence, this review identified the articles through a bibliographic search in two primary databases in this field, namely MEDLINE and PsycInfo. MEDLINE collects articles published in approximately 4500 biomedical reviews since 1966 from the USA and other 70 countries (US National Library of Medicine, 2021). PsycInfo database is the primary database of the American Psychological Association (APA) and the most important database in psychology worldwide (American Psychological Association [APA], 2021).

This bibliographic search was performed in January 2021 by using the Boolean operator ‘and’ and searching in all fields (field operator). The keywords used were ‘suicidal’, ‘ideation’ and ‘pregnancy’.

The complete electronic search strategy is presented in Table 1:

**Table 1:** Complete electronic search strategy

Databases	Keywords	Number of studies founded	Number of studies selected
Medline and PsycInfo	‘Suicidal’ [All Fields] AND ‘ideation’ [All Fields] AND ‘pregnancy’ [All Fields]	188	25

The search was limited to articles published in the last five years (between 2016 and 2021) in English and/or Spanish. The inclusion criteria included having a large sample and focusing on prevalence, risk factors, screening instruments, consequences or case management of suicidal ideation during pregnancy. The exclusion criteria included studies written in any language other than English or Spanish, published earlier than the last five years and focused on unrelated content. Articles with a sample size extremely small and not representative were excluded because those only referred to medical issues, non-psychiatric medication, periods other than pregnancy and those that even referred to suicidal constructs as not suicidal ideation.

Therefore, academic publications that addressed the most relevant and recent evidence on this topic were reviewed.

Selected articles met the SANRA scale criteria (Baethge et al., 2019). The articles’ quality was rated by using categories 0 and 2 to imply the low and high quality of each of the 25 studies, respectively, as shown in Table 2:

**Table 2:** Risk of bias of included articles in the narrative review

Study	SANRA items						Quality rating
	Item 1. Justification of the article's importance for the readership	Item 2. Statement of concrete aims or formulation of questions	Item 3. Description of the literature search	Item 4. Referencing	Item 5. Scientific reasoning	Item 6. Appropriate presentation of data	
Kalmbach et al., 2020	2	2	0	2	2	1	High (9)
Tabb et al., 2019	1	2	0	1	2	2	Medium (8)
Rodriguez et al., 2018	1	1	0	1	2	2	Medium (7)
Zhang et al., 2020	2	1	1	1	1	2	Medium (8)
O'Connor et al., 2018	1	2	2	2	1	1	High (9)
Levey et al., 2019	1	1	0	1	2	1	Medium (6)
Gelaye et al., 2016	2	2	2	1	1	2	High (10)
Onah et al., 2017	1	2	1	1	2	1	Medium (8)
Mebrahtu et al., 2020	2	1	1	1	1	1	Medium (7)
Rodriguez et al., 2017	1	1	1	1	1	1	Medium (6)
Chan et al., 2016	1	2	1	1	1	1	Medium (7)
Gelaye et al., 2019	1	1	1	1	1	1	Medium (6)
Shamu et al., 2016	1	1	1	1	2	2	Medium (8)
Mikšić et al., 2018	1	2	1	1	1	2	Medium (8)
Gelaye et al., 2017	1	1	1	1	1	2	Medium (7)
Zhong, Gelaye, et al., 2016	2	2	1	1	2	2	High (10)
Supraja et al., 2016	1	2	1	2	2	1	High (9)
Zewdu et al., 2021	1	1	1	1	2	2	Medium (8)
Luo et al., 2018	1	2	1	1	2	2	High (9)
Weng et al., 2016	1	2	1	1	2	2	High (9)
Musyimi et al., 2020	2	1	1	1	2	1	Medium (8)
Suzuki et al., 2019	1	2	2	1	2	2	High (10)
Palagini et al., 2019	1	2	1	1	2	2	High (9)
Zhong, Wells, et al., 2016	1	1	1	1	1	1	Medium (6)
Vergel et al., 2019	1	2	1	1	1	2	Medium (8)

Level at which each item is met 0 = low; 1 = medium; 2 = high. Quality of each article was determined based on the sum of its items 0 to 4 = low quality; 5 to 8 = medium quality; 9 to 12 = high quality.

## Results

A representative sample of the existent literature was summarized in a narrative synthesis, coupled with a critical overview of the current issues and questions that should be addressed within the topic of suicidal ideation during pregnancy. The available evidence was synthesized and critically discussed in the main challenges (issues and questions) in suicidal ideation during pregnancy which remains unaddressed, specifically regarding prevalence, risk factors, screening tools, consequences and case management.

**Fig. 1:**
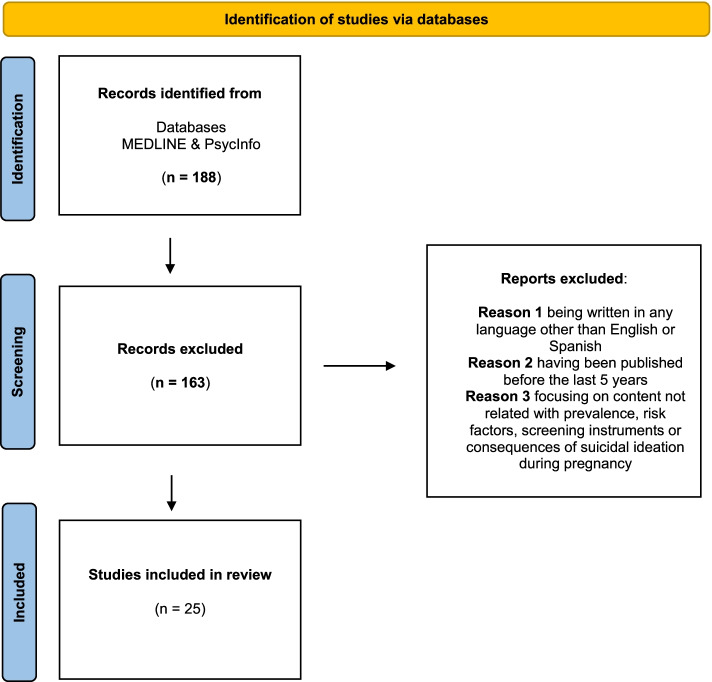
Flow diagram of the narrative review

From the 188 references found searching in MEDLINE and PsycInfo databases, 25 academic publications were selected, as shown in the flow diagram of this narrative review, adapted from PRISMA (which is indicated for systematic reviews; Page et al., 2021) (Fig. 1):

The primary topics were distributed, as shown in Table 3

**Table 3:** Topics covered in the selected articles on prenatal suicidal ideation

Topics on which they focus	Nº of studies
Risk factors	22
Prevalence	11
Evaluation instruments	5
Consequences	2

Most of the articles incorporate more than one theme

The highlights of the selected articles are shown in Table 4:

**Table 4:** Summary of reviewed studies on suicidal ideation during pregnancy

Author and year	Country and highlights
Kalmbach et al., 2020	USA Risk factors: insomnia and nocturnal rumination
Tabb et al., 2019	USA Prevalence: 4.6% Screening: EPDS
Rodriguez et al., 2018	South Africa Prevalence: 39% Risk factors: HIV, depression, intimate partner violence and younger age
Zhang et al., 2020	China Screening: PHQ-9 Risk factor: child physical abuse
O'Connor et al., 2018	International Risk factors: low socioeconomic background and intimate partner violence
Levey et al., 2019	Peru Risk factor: child abuse
Gelaye et al., 2016	International Risk factors: intimate partner violence, education less than 12 years and major depressive disorder
Onah et al., 2017	South Africa Prevalence: 18% Risk factors: major depressive episode, anxiety disorder, low socioeconomic status, food insecurity, interpersonal violence, multiparosity and previous suicide attempt throughout her life
Mebrahtu et al., 2020	Zimbabwe Screening: EPDS Risk factors: youth, singleness, hunger, high stress and symptoms of depression Consequence for the baby: worse cognitive development
Rodriguez et al., 2017	South Africa Prevalence: 39% Risk factors: HIV, stigma and intimate partner violence
Chan et al., 2016	Malaysia Risk factor: religious belief that premarital sex is wrong
Gelaye et al., 2019	Peru Prevalence: 8.7% Consequence for the baby: low birth weight
Shamu et al., 2016	Zimbabwe Risk factor: emotional partner violence
Mikšić et al., 2018	Croatia Prevalence: 2.73% Risk factors: anxiety and depression
Gelaye et al., 2017	Peru Screening: PHQ-9 Prevalence: 8.5% Risk factor: poor quality of sleep
Zhong, Gelaye, et al., 2016	USA Risk factors: be black and have low income
Supraja et al., 2016	India Risk factors: youth, poor perceived support, intimate partner violence, depressive symptoms and history of suicide attempt
Zewdu et al., 2021	Ethiopia Prevalence: 8.2% Risk factors: depression, undisclosed HIV status and unwanted pregnancy
Luo et al., 2018	China Prevalence: 8.2% Risk factor: previous induced abortion
Weng et al., 2016	China Risk factor: passive exposure to tobacco
Musyimi et al., 2020	Kenya Risk factors: low economic status and intimate partner violence
Suzuki et al., 2019	International Risk factors: passive exposure to tobacco
Palagini et al., 2019	Italy Risk factor: stress-related sleep difficulties
Zhong, Wells, et al., 2016	Peru Prevalence: 15.8% Screening: PHQ-9 Risk factors: physical or sexual abuse during childhood
Vergel et al., 2019	Colombia Prevalence: 7.2 Risk factors: obstetric risk during first trimester of pregnancy

### Prevalence of suicidal ideation during pregnancy

Pregnant women are more likely than the general population to present suicidal ideation (Gelaye et al., 2016).

The selected studies provided data on the prevalence of suicidal ideation which is 2.73% in pregnant Croatian women (Mikšić et al., 2018), 4.6% in pregnant American women (Tabb et al., 2019) and 8.2% in pregnant Chinese women (Luo et al., 2018).

A higher prevalence of suicidal ideation was found in African women than in pregnant Western women. Onah et al. (2017) found that the prevalence of suicidal ideation during pregnancy is 18% in South African women. This number increases up to 39% if they have human immunodeficiency virus (HIV; Rodriguez et al., 2018; Rodriguez et al., 2017). However, in Ethiopian women with HIV, the prevalence of suicidal ideation is low (8.2%; Zewdu et al., 2021).

The prevalence rates of pregnant Latina women are also high. In pregnant Peruvian women, the prevalence rate is between 8.5% and 15.8% (Gelaye et al., 2017; Gelaye et al., 2019; Zhong, Wells, et al., 2016). Meanwhile, in pregnant Colombian women, the prevalence of suicidal ideation is 7.2% (Vergel et al., 2019).

### Risk factors for suicidal ideation during pregnancy

Most studies focused on this topic.

One risk factor is clinical conditions. Pregnant women with suicidal thoughts are more anxious and depressed (Mikšić et al., 2018). Major depressive disorder is an identified risk factor (Gelaye et al., 2016). Depressive symptoms and a history of suicide attempts are predictive factors (Supraja et al., 2016). Having a major depressive episode, an anxiety disorder or a previous suicide attempt throughout life are also risk factors (Onah et al., 2017). Mebrahtu et al. (2020) also identified high stress and symptoms of depression as risk factors. Pregnant women with insomnia and high rumination have higher rates of suicidal ideation than the general population (Kalmbach et al., 2020). Pregnant women with sleep difficulties have a higher chance of having suicidal ideation than those without sleep difficulties (Palagini et al., 2019). Poor sleep quality is associated with a 2.81-fold increased likelihood (Gelaye et al., 2017).

The second factor is social inequalities. Suicidal ideation amongst pregnant women is higher amongst blacks than amongst whites in those with low incomes (Zhong, Gelaye, et al., 2016). In a systematic review, Gelaye et al. (2016) identified experiencing intimate partner violence and having fewer than 12 years of education as risk factors. Young age, poor perceived support and partner violence are also predictable factors (Rodriguez et al., 2018; Supraja et al., 2016). A low socioeconomic level, suffering food insecurity and interpersonal violence are also risk factors (Onah et al., 2017). Shamu et al. (2016) also identified emotional violence from the partner (which had a greater effect than physical or sexual violence) as a risk. Musyimi et al. (2020) also identified low economic status and intimate partner violence as consistently associated factors. Women who reported suicidal ideation tend to be young, single and experienced hunger (Mebrahtu et al., 2020). The religious belief that premarital sex is wrong is also related to a high risk of suicidal ideation (Chan et al., 2016).

The third factor is previous traumatic situations. Child abuse increases the risk of suicidal ideation during pregnancy by 2.57- (Levey et al., 2019) to 2.9-fold (Zhong, Wells, et al., 2016). The risk increases as the number of experienced child abuse events increases (Zhong, Wells, et al., 2016). Different studies showed that pregnant women who have suffered from physical abuse during childhood have a high risk of suicidal ideation (Zhang et al., 2020).

The fourth factor is gestation. Women hospitalized for high obstetric risk have suicidal ideation (Vergel et al., 2019). The prevalence of suicidal ideation increases almost twofold if pregnant women have suffered an induced abortion during that year (especially if they were also single; Luo et al., 2018). Suicidal ideation is also associated with multiparity (Onah et al., 2017).

The last risk factor is other health circumstances, such as passive exposure to tobacco (Suzuki et al., 2019; Weng et al., 2016) and HIV (Rodriguez et al., 2017;Rodriguez et al., 2018 ; Zewdu et al., 2021).

### Screening tools for suicidal ideation during pregnancy

Researchers used either item 10 of the Edinburgh Postnatal Depression Scale (EPDS) (Mebrahtu et al., 2020; Tabb et al., 2019) or item 9 of the Patient Health Questionnaire (PHQ-9; Gelaye et al., 2017, 2019; Zhang et al., 2020; Zhong, Wells, et al., 2016) to detect suicidal ideation in pregnant women.

### Consequences of suicidal ideation during pregnancy

Few studies focused on the consequences of suicidal ideation during pregnancy. However, those published studies provided relevant conclusions. For example, a study reported that suicidal ideation is associated with poor cognitive development in children (Mebrahtu et al., 2020). Another study showed that pregnant participants with suicidal ideation have a fourfold risk of delivering a low-birth-weight baby (Gelaye et al., 2019).

## Discussion

This narrative review synthesized the state-of-the-art knowledge regarding prevalence, risk factors, screening tools, consequences and case management. This paper contributed to identifying several important questions that remain to be answered concerning these issues. This research showed international evidence about suicidal ideation during pregnancy in European, American, African, Asian and Latin American women.

Baumeister and Leary (1997) suggested that in discussing narrative reviews (section critiques instead of criticizing each study), each section may involve a summary of the methods and results of a group of studies relevant to a point and a brief outline of major flaws of that evidence.

Available evidence showed that pregnant women are more likely to have suicidal ideation than the general population (Gelaye et al., 2016), oscillating around 2.73% in European countries (Mikšić et al., 2018), 8.5–15.8% in Latin American countries (Gelaye et al., 2017; Gelaye et al., 2019; Zhong, Wells, et al., 2016) and 39% in African women (Rodriguez et al., 2018; Zewdu et al., 2021). Pregnancy is when women are particularly vulnerable to mental health problems, including suicidal ideation (Szpunar et al., 2020). Several studies in the narrative review confirmed this result which showed that suicidal ideation is higher in pregnant women than in the general population (Gelaye et al., 2016; Rodriguez et al., 2018).

Secondly, this narrative review identified specific risk factors for suicidal ideation during pregnancy. According to O'Connor et al. (2018), knowing vulnerability factors allows the close monitoring of pregnant women for suicidal ideation and contributes to the necessary prevention of pregnancy-related suicidal behaviour. The well-established risk factors for suicidal ideation during pregnancy include depressive disorder, less than 12 years of education and intimate partner violence (Gelaye et al., 2016). Other risk factors are anxiety (Mikšić et al., 2018), child abuse (Levey et al., 2019; Zhang et al., 2020; Zhong, Wells, et al., 2016), previous suicide attempts (Onah et al., 2017; Supraja et al., 2016), insomnia (Gelaye et al., 2017; Kalmbach et al., 2020; Palagini et al., 2019), low income (Musyimi et al., 2020; O'Connor et al., 2018; Zhong, Gelaye, et al., 2016), poor perceived support (Supraja et al., 2016), tobacco consumption (Suzuki et al., 2019; Weng et al., 2016), obstetric risk (Vergel et al., 2019) and induced abortion (Luo et al., 2018). Knowing the risk factors for suicidal ideation during pregnancy is useful to prevent possible associated suicidal behaviour. This result aligns with the COPE which developed a guideline on mental healthcare during the perinatal period that considers suicide risk (Centre of Perinatal Excellence [COPE]., 2017).

Thirdly, regarding the screening, we found that researchers used either item 10 of the EPDS (Mebrahtu et al., 2020; Tabb et al., 2019) or item 9 of the PHQ-9 (Gelaye et al., 2017, 2019; Zhang et al., 2020; Zhong, Wells, et al., 2016). This result was consistent with what was known about general suicidal ideation assessment (Al-Halabi et al., 2019). Zhong et al. (2015) showed that the PHQ-9 questionnaire is more sensitive than the EPDS as a screening tool for suicidal ideation amongst pregnant women.

In the narrative review, studies about the consequences of suicidal ideation during pregnancy were scarce. Suicidal ideation during pregnancy was associated with adverse consequences, such as poor child's cognitive development (Mebrahtu et al., 2020) and a high probability of giving birth to a baby with low birth weight (Gelaye et al., 2019). Previously available literature also pointed out that antepartum suicidal ideation is associated with adverse infant outcomes (Copersino et al., 2008; Gavin et al., 2011; Lindahl et al., 2005). This narrative review also showed few studies on the consequences of suicidal ideation during pregnancy, such as emotional issues or attachment; effects on mothers themselves, their partners, family members and friends and cost-effectiveness. However, the consequences of suicidal ideation during pregnancy generate social and health costs (Al-Halabí, 2019a). Further work on this topic is highly necessary.

Lastly, we expected to find various studies on the case management of suicidal ideation in pregnant women. However, no article in the narrative review addressed this issue, despite all the evidence describing the risk of suicidal behaviour during gestation.

Hence, various clarifications are needed. The prevalence of suicidal ideation during pregnancy is between 2.3 and 2.7% internationally (Arachchi et al., 2019). The high rates are related to the peculiarities of the samples, the cultures and assessment methods. Pregnancy itself or the act of having a child are also protective (and not risk) factors for suicidal behaviour (Al-Halabí et al., 2019b). Despite suicidal ideation being frequent during pregnancy, suicidal behaviour is less frequent than in the general women population (Lysell et al., 2018).

However, although suicidal behaviour during pregnancy does not have a high prevalence compared with the rate in the general population, the consequences for families and children who lose their mothers show the need for preventive actions (Lysell et al., 2018). Hence, the risk factors found in this narrative review should be considered. The development of effective case management procedures is also essential. As Musyimi et al. (2020) highlighted, family members, community leaders, healthcare workers and policymakers should explore ways to manage suicidal ideation during pregnancy. The need for training in suicide for professionals involved in perinatal care and collaboration amongst gynaecologists, paediatricians and psychologists was also demonstrated in the study of Rodríguez-Muñoz et al. (2019). This multidisciplinary approach would contribute to the much-needed prevention of suicide, a public health problem.

The main limitation of the available studies was the lack of articles with a high degree of methodological rigour on this subject. Studies found about the management of suicidal ideation in pregnant women were also scarce. Notably, the implementation of studies that reduce suicide was completely economically profitable for our healthcare system because suicidal behaviour contributes, amongst other adverse consequences, to early death, morbidity, loss of productivity and increased costs of medical care (Hughes, 2020).

Gelaye et al. (2016) noted that the antepartum period represents a critical period and an important opportunity to reduce and prevent suicide risk. Access to clinical interventions and support in seeking professional help are protective factors for suicide associated with pregnancy (Turecki & Brent, 2016). Hence, studies on case management are needed. We have evidence of the need for evidence-based interventions for managing suicidal ideation amongst pregnant women.

Despite the non-systematic nature of this review, the synthesis allows us to reflect on the critical challenges and questions that should be addressed within the scope of maternal suicidal ideation research and clinical practice. Overall, narrative reviews, systematic reviews and meta-analyses should be performed to include their evidence in clinical practise guidelines and protocols. This effort would improve our evidence-based practice in suicidal ideation during pregnancy.

## Conclusions

A comprehensive understanding of maternal suicidal ideation is necessary to answer crucial questions regarding the issue. To our knowledge, this work is the first comprehensive one on suicidal ideation during pregnancy.

Making research efforts that contribute to the identification and case management of pregnant women at risk of suicidal ideation is important. Screening and based-evidence protocols should be developed for better care practice with pregnant women. This issue is urgent and should be addressed immediately.

This narrative review is a state-of-the-art paper about suicidal ideation during pregnancy. We hope that this work will advance this limited field of knowledge. Research on suicidal ideation during pregnancy should contribute to clinical evidence-based practice.

Future research should address crucial questions regarding the prevention, identification and management of suicidal ideation (Al-Halabí & Fonseca-Pedrero, 2021) during this period to prevent maternal suicidal ideation consequences (Al-Halabí et al., 2021).
